# Development of a Novel Two Dimensional Surface Plasmon Resonance Sensor Using Multiplied Beam Splitting Optics

**DOI:** 10.3390/s130100801

**Published:** 2013-01-08

**Authors:** Akihide Hemmi, Ryosuke Mizumura, Ryuta Kawanishi, Hizuru Nakajima, Hulie Zeng, Katsumi Uchiyama, Noriaki Kaneki, Toshihiko Imato

**Affiliations:** 1 Mebius Advanced Technology Ltd., 3-31-6-107 Nishiogi-kita, Suginami-ku, Tokyo 167-0042, Japan; 2 Department of Applied Chemistry, Graduate School of Engineering, Kyushu University, 744 Motooka, Nishi-ku, Fukuoka 819-0395, Japan; 3 Department of Applied Chemistry, Graduate School of Urban Environmental Sciences, Tokyo Metropolitan University, 1-1 Minamiohsawa, Hachioji, Tokyo 192-0397, Japan; E-Mails: mizumura-ryosuke@ed.tmu.ac.jp (R.M.); ryuta0120@gmail.com (R.K.); zeng-hulie@tmu.ac.jp (H.Z.); uchiyama-katsumi@tmu.ac.jp (K.U.); 4 College of Design and Manufacturing Technology, Graduate School of Engineering, Muroran Institute of Technology, 27-1 Mizumotocho, Muroran 050-8585, Japan; E-Mail: kaneki@epsilon2.csse.muroran-it.ac.jp

**Keywords:** two dimensional sensor system, multichannel, surface plasmon resonance, sensor

## Abstract

A novel two dimensional surface plasmon resonance (SPR) sensor system with a multi-point sensing region is described. The use of multiplied beam splitting optics, as a core technology, permitted multi-point sensing to be achieved. This system was capable of simultaneously measuring nine sensing points. Calibration curves for sucrose obtained on nine sensing points were linear in the range of 0–10% with a correlation factor of 0.996–0.998 with a relative standard deviation of 0.090–4.0%. The detection limits defined as S/N = 3 were 1.98 × 10^−6^–3.91 × 10^−5^ RIU. This sensitivity is comparable to that of conventional SPR sensors.

## 1. Introduction

SPR is one of the most powerful analytical methods for evaluating biomolecular interactions in real-time without the need for labeling. Using SPR, it is possible to measure changes in refractive index on the small and thin area of the sensor chip surface. Because of these benefits, SPR sensors have been widely used for detecting various chemical and biological compounds in the laboratory in the fields of environmental analysis [1], food safety [2], and clinical diagnostics [3].

It is noteworthy that the use of SPR sensor systems has been undergoing a change. For example, in novel drug projects in the field of pharmaceutical biotechnology, a compact desktop type SPR sensor system with a higher throughput would be desirable [4]. Also, in the field of social healthcare, as a safety countermeasure for the spreading of infection diseases, a handheld type SPR sensor system for on-site analysis is urgently needed [5].

In order to comply with these needs, an SPR sensor system with higher throughput with multi-channel sensing points and a more compact size is desired. In the case of a conventional type SPR sensor system, which is comprised of a Kretschmann configuration system [6], the optics system of the instrument can be classified into one of three types:

The first type employs a fan shaped mono-chromatic beam as the surface plasmon incident light source with a hemi-circular prism as the surface plasmon coupler [7]. As a detector in this optic system, a linear CCD sensor or a 2D CCD camera is usually used to measure the intensity of the reflected fan shaped light, including the SPR dip. The SPR sensor response is measured as a change in the SPR dip position. The advantage of this system is that it permits the SPR response to be observed over a wide range of approximately 10 degrees at a time and that it does not contain any moving parts like a motor stage for scanning angles.

The second type uses a particular fixed angle small point mono-chromatic beam as the surface plasmon incident light source with a triangular or a hemi-circular prism as the surface plasmon coupler [8]. The angle is set near the expected SPR dip. A photodiode is usually used in this system to measure the intensity of the reflected light at the same fixed angle of the incident light. Since the SPR sensor signal is observed as the intensity of the reflected light at the shoulder of the SPR sensor response curve, the sensitivity is higher than that for measuring the change in the SPR dip angle. However, many samples cannot be measured using this system, because the sensing region is only one point. Also, the system needs stages for scanning the dip position of the SPR sensor curve to determine the initial observing angle before the measurement.

The third type is a combination of the optic systems discussed above. An angle scanning mono-chromatic beam is used as an incident light to form a fan-like shaped beam. The incident light is achieved using a galvanometer based optical scanner, or by moving the position of the beam along the center of the sensing point of the prism surface using a rotary stage [9]. To measure the intensity of the reflected light along the scanning angle, a photodiode which is able to cover the angle range of the reflected light or a 2D CCD camera is usually used as the detector. The SPR sensor signal response is measured as the change in the SPR dip position because the system is able to observe SPR sensor curve.

In general, these SPR optical configurations have a point or a line shaped sensing region that is approximately 10 to 20 mm in length and 100 μm wide on a sensor chip. In order to achieve a higher throughput measurement system, it is necessary to have a number of flow channels so that multiple samples can be measured simultaneously, and a high speed sample changing fluid system. However, the number of flow cell channels that can be placed on the sensing region for multi-sensing are limited to 20 channels because a flow cell cannot be placed in narrow line shaped sensing region.

On the other hand, another approach to resolving these issues is an SPR imaging system [10–15]. The system was designed to observe a certain area on the sensor chip. The size of the sensing area is approximately 20 mm square. To simultaneously measure a number of samples, the sample cell size is usually set to a square with sides of approximately 100 μm in the sensing area. However, there are difficulties associated with the fabrication of such microscopic cells. Therefore, additional sensor chip preparation equipment, like a material spotting inkjet printer, is needed for setting different kinds of samples on microscopic cells. A large circular collimated light is also required to irradiate the wide sensing area on the prism. For that purpose, a beam expander is usually used to reshape the small beam spot size of the laser diode into a large collimated light. The optical pathlength of the system become long. The system needs a more expensive high resolution format CCD camera compared to other SPR systems.

These conventional SPR systems are almost desktop in size. Considering the fact that they are used for on-site analysis, they may not be suitable, because of the size, weight and cost of the instrumentation.

In previous studies, we reported on the development of portable SPR sensor optics and sensor chips with dual- [16,17] or multi- [18] detection points, which are very compact in size relative to the optics and sensor chips. They showed good sensitivity as conventional desktop type SPR sensor systems.

In this study, we wish to report on the development of a novel two dimensional SPR sensor system with a nine point sensing region on the sensor chip at intervals of *ca.* 5 mm in a reticular pattern. A multiplied beam splitting optics for achieving multi-point sensing was newly designed and fabricated. The system has a scalable optical configuration, which permits the number of sensing points to be increased, with no lens. Therefore, the system is applicable to on-site analysis applications mentioned above.

## 2. Experimental Section

### 2.1. Materials and Reagents

Polydimethylsiloxane (PDMS) prepolymer (Sylpot 184), and its curing agent were obtained from Dow Corning Toray Co., Ltd. (Tokyo, Japan). Sucrose was obtained from Wako Pure Chemical Industries, Ltd. (Osaka, Japan). All dilution steps were performed with Millipore filtered water from a Milli-Q system (Nihon Millipore, Tokyo, Japan).

### 2.2. Fabrication and Principle of the Multiple Beam Splitter Light Source

**Figure 1.**
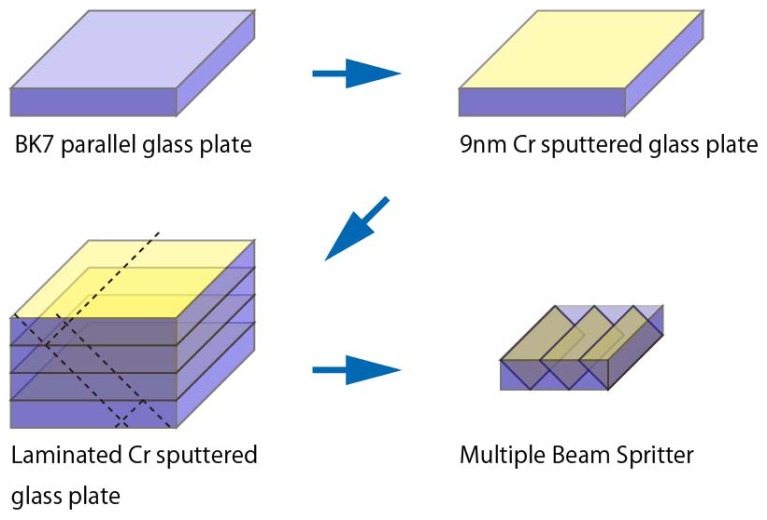
Method of fabrication for multiple beam splitter optics.

Figure 1 shows the method used in the fabrication of the Multiple Beam Splitter Optics used in this study. First, as a reflection film of the beam splitter, thin metal chromium was deposited on a BK7 parallel glass plate (12 (width) × 18 (height) × 5 (thickness) mm, KADOMI Optical Industry, Tokyo, Japan) with a thickness of *ca.* 9 nm using sputtering equipment (M92-0007, SEED Lab. Co., Kanagawa, Japan). The three sputtered glass plates were then laminated horizontally using a UV optical adhesive (PHOTOBOND 400, Sunrise MSI Co., Osaka, Japan), so that each of the three reflection film planes were parallel. The block of a laminated beam splitter glass plate was formed into a size of 12 (Width) × 18 (Height) × 5 (thickness) mm, and the reflection angle of film plane was maintained at 45 degrees to the beam entrance surface.

**Figure 2.**
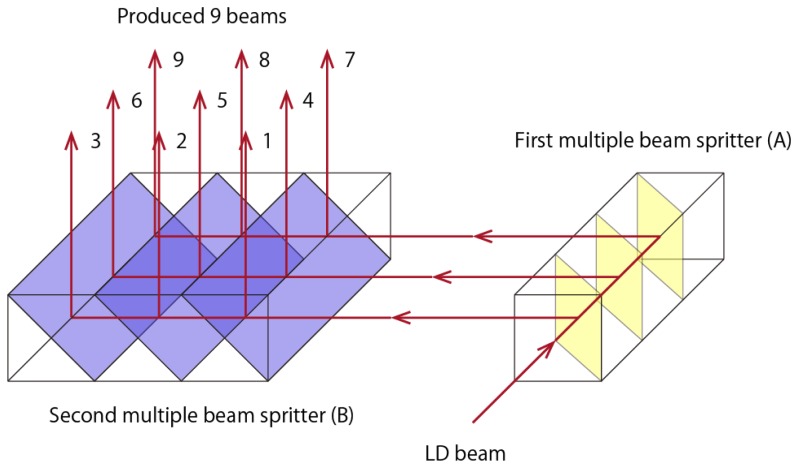
Perspective diagram of direction of radiation of the laser beam.

Figure 2 shows the perspective diagram of the direction of radiation of the laser beam. A laser diode module (655 nm, 350 μW, LV-S41, KEYENCE, Osaka, Japan) was used as the light source. In order to transform one beam to nine beams, two multiple beam splitters were arranged in rows and columns. At the first multiple beam splitter, denoted as A in Figure 2, a laser beam light source was entered and transformed into the three beams on the three reflecting films. Each divided parallel light beam was irradiated onto the second multiple beam splitters, denoted as B in Figure 2. As a result, nine, two dimensional, parallel light beams were observed at the exit of the second multiple beam splitters. Each of the nine beams was assigned a number for convenience as shown in Figure 2. The intervals of the nine beams were 5 mm each and the shape of the beams was the same. The power of each beam was measured using a handy laser power meter (7Z01560, Ophir Optronics Solutions Ltd., Jerusalem, Israel). The calculated reflectivity, transparency and absorption factor of P-polarized and S-Polarized light using the optical design software, ZEMAX (Radiant Zemax LLC, Redmond, WA, USA) is shown in Table 1.

Table 2 shows the calculated power of the nine beams with the optical factor in Table 1. The normalized observed power of each beam was correlated well with the calculated powers.

### 2.3. Optical System of 2D SPR Sensor

**Figure 3.**
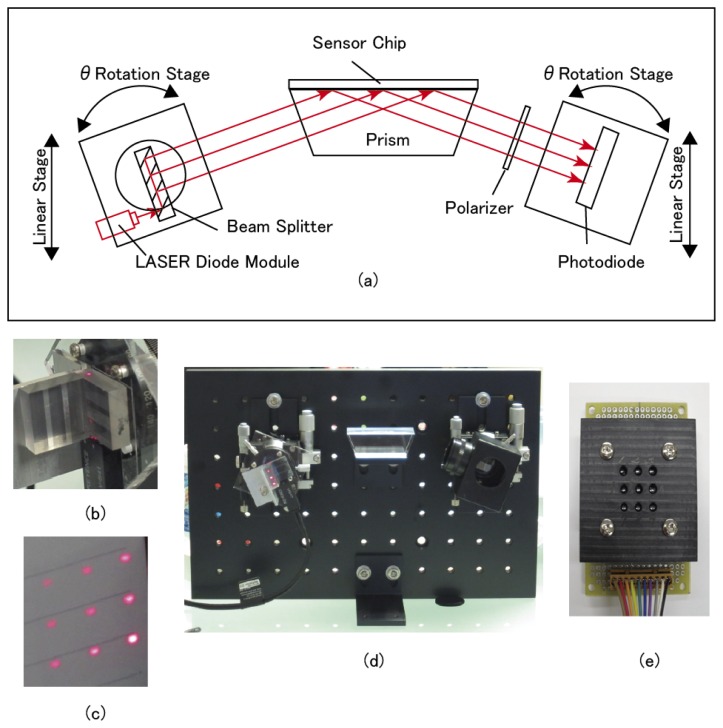
(**a**) Optical System for the 2D SPR Sensor. (**b**) Multiplied beam splitter with LD light source. (**c**) Nine beams projected on the paper at the exit of the multiplied beam splitter. (**d**) Photo of optical system. (**e**) Arrayed photo diode detector.

Figure 3 shows the optical system for the 2D SPR sensor developed in this study. The configuration of the optical system and the theory of its operation were based on the principle of a conventional fixed angle light intensity type SPR sensor system, except for the multiple beam splitter light source.

The optical system was constructed on an aluminum optical base plate (BBA6-200-300, 300 mm in width × 200 mm in height, MISUMI Corporation, Tokyo, Japan). The light source, the multiplied nine laser beams optic, was set on a rotary stage (RPG38, MISUMI Corporation) as the *θ*-Axis transfer and a linear stage (XCRS40, MISUMI Corporation) as the Y-Axis transfer. The detector consisted of a polarizer (NT47-315, Edmund Optics Inc., Barrington, NJ, USA) and nine photodiodes (SP-1CL3, KODENSHI CORP., Kyoto, Japan) set in a lattice pattern. The lattice pattern interval is the same as the multiplied nine laser beams. These were set on a rotary stage (RPG38, MISUMI Corporation) as the *θ*-Axis transfer and a linear stage (XCRS40, MISUMI Corporation) as the Y-Axis transfer. A trapezoidal prism (BK7, 54 mm (upper side) × 39.7 mm (lower side) × 18.7 mm (height) × 30 mm (width), 69° base angle, KADOMI Optical Industry, Tokyo, Japan) was centrally located between the light source and the detector. An optically parallel glass plate (BK7, surface flatness of 2λ, parallelism of 60″, 54 mm distance × 30 mm width × 0.5 mm thickness), sputtered with a 3 nm layer of titanium and a 45 nm layer of gold using sputtering equipment (M92-0007, SEED Lab. Co., Kanagawa, Japan), was used as the SPR sensor chip. The Au sensor chip was optically integrated with the trapezoidal prism surface using matching oil (n_D_ = 1.5150 ± 0.0002, Type A, Catalog No. 16482, Cargille Laboratories Inc., Cedar Grove, NJ, USA).

The light beams from the multiple beam splitter optics entered the trapezoidal prism directly. The center beam of the nine beams was set to the center of the surface of the Au sensor chip. The reflected light beams were then passed through the polarizer to eliminate s-polarized light. The detector was set so that the each beam reached the corresponding photodiode element respectively.

**Figure 4.**
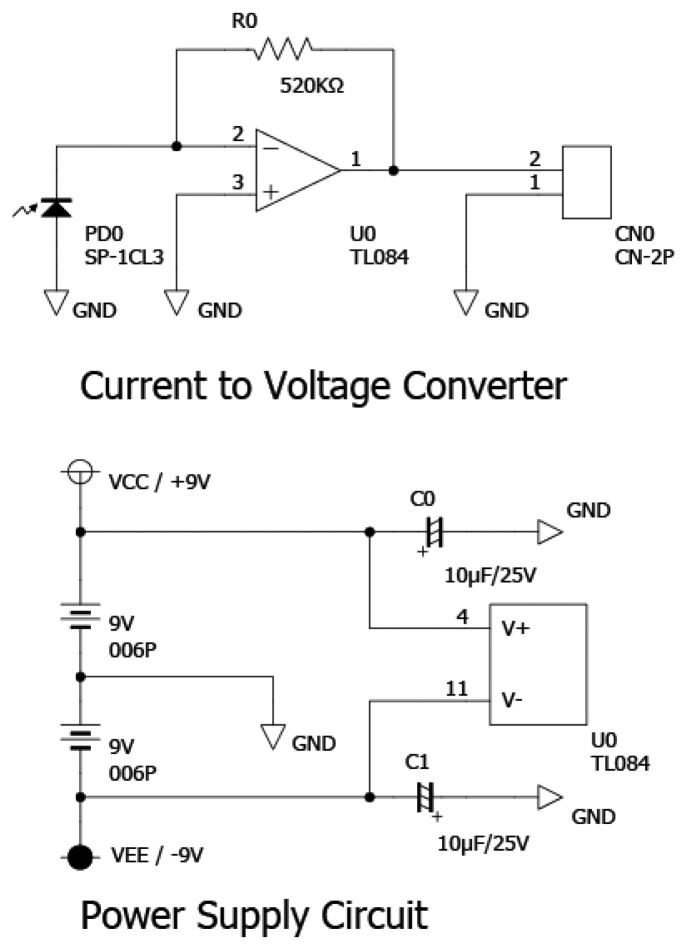
Schematic diagram of the current to voltage circuit.

The working distance between the center of the prism and the center of light beam source was 94 mm at 70 degrees of incident light. The p-polarized light of the reflected light corresponding to the SPR was measured under dark room conditions. The signal of the SPR sensor was obtained by subtracting the dark current signal from the observed signal. The photocurrent from each photodiode was converted to voltage by current to voltage circuit (Figure 4). The each voltage was recorded using 34970A Data Acquisition/Data Logger Switch Unit (Agilent, Loveland, CO, USA) with a 20 Channel Multiplexer Module 34901A (Agilent) and a BenchLink Data Logger software (Agilent).

### 2.4. Fabrication of the Sensor Chip Fluidics

**Figure 5.**
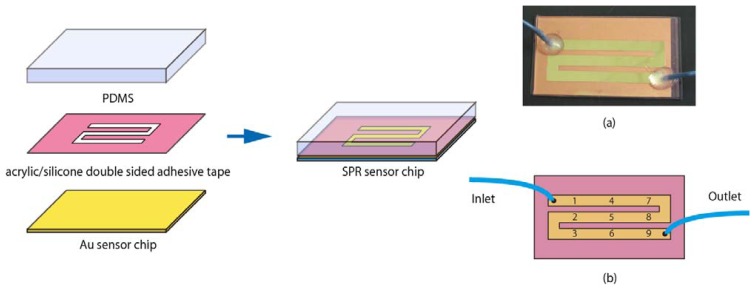
Fabrication of the sensor chip fluidics. (**a**) Photo of sensor chip. (**b**) The detection point in the flow cell. The numbers correspond to the beam number.

First, as shown in Figure 5, an A4 formatted sheet of acrylic/silicone double sided adhesive tape obtained from Sumitomo 3M Limited Company (No. 4357, Tokyo, Japan) was cut out to form a flow cell. The flow channel size was 4 mm in width, 136 mm in length with a thickness of 0.1mm. The flow channel sheet was then set on the Au sensor chip with the adhesive. The Au sensor chip with the flow channel was covered with a PDMS plate (54 mm width × 30 mm distance, thickness of 5 mm) with two 1.5 mm diameter holes as an inlet and an outlet port. Finally, two PEEK tubes (I.D. 0.25 mm, O.D. 1/16 inches) were connected into inlet and outlet port using adhesive (SuperX No. 8008, CEMEDINE CO. LTD., Tokyo, Japan) for sending the sample solutions. The volume of the flow cell was 54.4 μL.

### 2.5. Flow Analysis System Using 2D SPR Sensor

**Figure 6.**
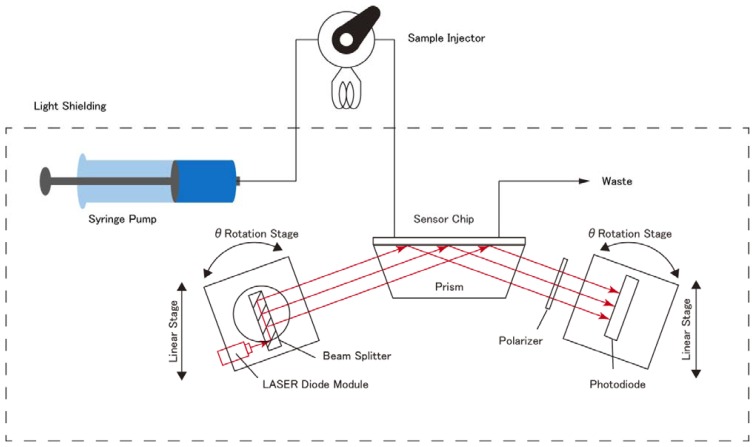
Flow analysis system for 2D SPR sensor.

Figure 6 shows the 2D SPR Sensor system. The system consisted of a syringe pump (PHD 2000 Infusion 70-2000, Harvard Apparatus, Holliston, MA, USA), a sample injector (VI-11, FLOM Co. Ltd., Tokyo, Japan) with a sample loop of 150 μL, the sensor chip, and the developed 2D SPR sensor.

## 3. Results and Discussion

### 3.1. 2D SPR Sensor Response for the Water Sample

**Figure 7.**
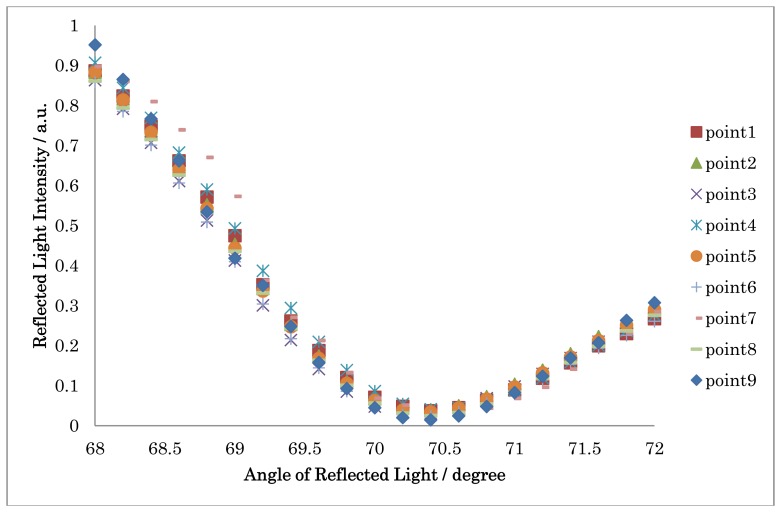
The SPR curves for nine measurement points.

In the case of water based sample solution measurement, the system must be set for a specific initial incident light angle position before the measurement. To seek the angle, the SPR sensor response for an aqueous sample from 68 to 72 degrees was measured in 0.2 degree steps. For angle position settings, the position of the stages was set using scale marks stamped on the stages. First, 20 μL of matching oil was placed on the prism surface. The sensor chip fluidics were then placed on the prism surface tightly and filled with water. The SPR curves obtained on nine measurement points are shown in Figure 7. The dips of the SPR curves were observed at around 70.4 degree. To measure the change in the reflected light intensity based on the change in refractive index, the angle was fixed at 69.8 degree for measurement of the water based sample solution.

### 3.2. Evaluation of Basic Performance of the 2D SPR Sensor

**Figure 8.**
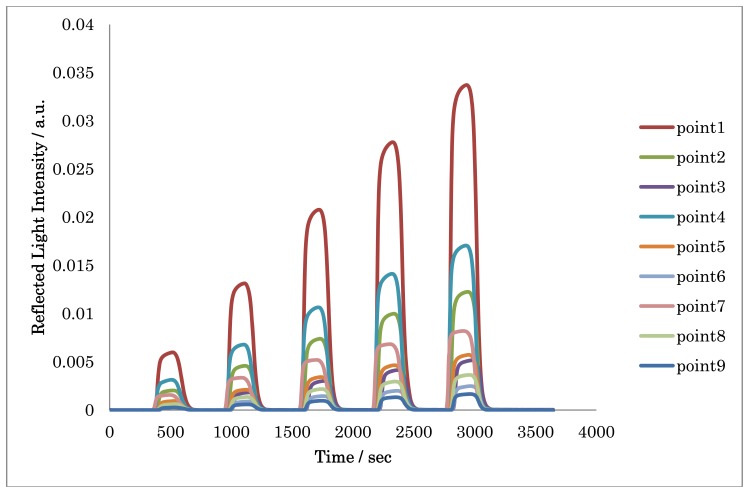
The 2D SPR sensorgrams for sucrose solutions.

The basic performance of the 2D SPR sensor was evaluated using several concentrations of sucrose solutions. To measure the background light intensity, water was allowed to flow at rate of 50 μL/min for 5 min. Then, 150 μL of sucrose solutions, the concentrations of which were from 2 to 10 wt%, were sequentially injected at 10 min intervals. Figure 8 shows the nine sample point sensorgrams for sucrose solutions. The intensity of the reflected light increased with increasing the concentration of sucrose solution.

The nine sensorgrams cannot be compared with each other in terms of raw data, since the intensity of the laser beam from the multiplied beam splitter is different. Therefore, to prepare a calibration curve, the calibrated intensity data of nine sensorgrams is required. The intensity of the calibrated data (*I_Calib_*) was calculated by the following Equation (1):

(1) $$I_{\text{Calib}}=\frac{\left(I_{\text{Suc}})-(I_{\text{Water}}\right)}{\left(I_{\text{Air}})-(I_{\text{Blank}}\right)}$$

where, *I_Suc_* is the reflected light intensity of the sucrose solution, *I_Air_* is the reflected light intensity of the total reflection with air, *I_Water_* is the reflected light intensity of the water and *I_Blank_* is the reflected light intensity without light.

**Figure 9.**
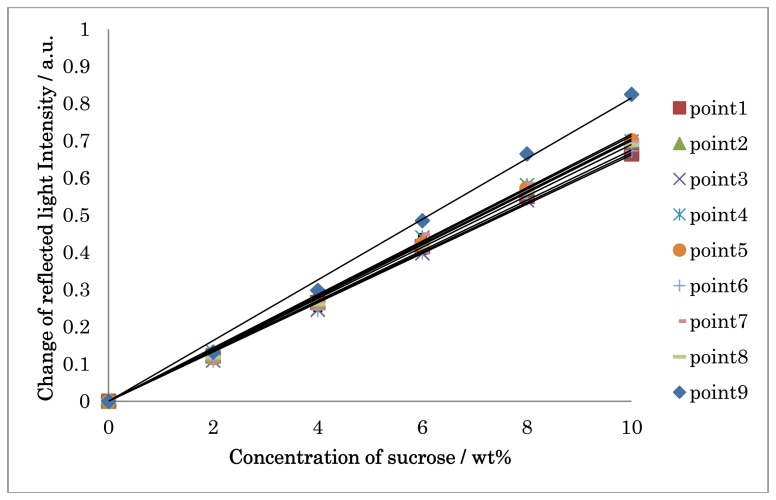
Calibration curve for sucrose on nine sensing points.

Figure 9 shows the calibration curve for sucrose on nine sensing points with the calibrated intensity values. The linearity in the range of 0–10% with the correlation factor of 0.996–0.998 and a relative standard deviation of 0.090–4.0% were obtained. The detection limits, defined as S/N = 3, were 1.98 × 10^−6^–3.91 × 10^−5^ RIU. This sensitivity of the developed system is comparable to that of our previous study [14].

## 4. Conclusions/Outlook

The optical system developed in this study is superior to other systems in scalability because it is comprised of a simple optical configuration without a lens. The size of all of the optical elements from the light source to the prism and detector can be readily changed by scaling-up or scaling-down. This feature can be realized by changing the size of the trapezoidal prism for sample installation, the number of multiplied laser beams, the thickness of sputtered chromium for the light intensity of each beam, the intervals of the beams and the number of detectors.

When the optical elements are miniaturized, it becomes possible to manufacture a palm-sized multichannel SPR system using micro-sample cells. When the optical elements are enlarged, a SPR system using a 96-well microtiter plate as a measurement cell can be manufactured. Since no lens is used in this SPR sensor system for the convergence of light paths, the manufacturing costs can be expected to be modest and the size of the system would be compact compared to the conventional systems used currently.

## Figures and Tables

**Table 1.** Calculated optical factor of the beam splitter.

	**S polarization**	**P polarization**
Reflectance	0.392	0.182
Transmittance	0.330	0.575
Absorbance	0.278	0.243

**Table 2.** Observed powers of the nine beams.

**Point of beam**	**Calculated power**	**Observed power (nW) (raw data)**	**Observed power (normalized data)**
1	100	54.3	100
2	33.0	19.7	36.3
3	10.9	9.12	16.8
4	57.5	25.7	47.3
5	19.0	10.1	18.6
6	6.27	5.18	9.54
7	33.1	16.2	29.8
8	10.9	6.44	11.9
9	3.61	3.56	6.56
